# A new species of the genus *Chinattus* Logunov, 1999 from Wuyi Mountain National Park, China (Araneae, Salticidae)

**DOI:** 10.3897/BDJ.13.e155002

**Published:** 2025-05-29

**Authors:** Xiaoting Lyu, Qiangyong Fan, Zihan Cai, Zhian Zou, Keke Liu, Binlu Liu

**Affiliations:** 1 Key Laboratory of Jiangxi Province for Biological Invasion and Biosecurity, Jinggangshan University, Ji'an, China Key Laboratory of Jiangxi Province for Biological Invasion and Biosecurity, Jinggangshan University Ji'an China; 2 Jiangxi Administration Bureau, Wuyi Mountain National Park, Shangrao, China Jiangxi Administration Bureau, Wuyi Mountain National Park Shangrao China

**Keywords:** Jiangxi Province, Jumping spiders, new species, taxonomy

## Abstract

**Background:**

The genus *Chinattus* Logunov, 1999, a group of jumping spiders (Salticidae), has been recorded across multiple regions in China. Current records indicate its presence in Anhui, Hunan, Yunnan, Hubei, Taiwan, Guangdong, Hong Kong, Guizhou, Fujian, Chongqing and Sichuan Provinces. These reports highlight a fragmented, but widespread distribution in southern and central China, particularly in mountainous or subtropical habitats. Notably, Jiangxi Province, situated adjacent to several documented regions (e.g. Hunan, Fujian), lacks any confirmed records of *Chinattus* to date. This absence raises questions about potential sampling gaps, ecological barriers or environmental factors limiting its presence in Jiangxi, warranting further investigation to clarify the genus' full geographic range and habitat preferences within China.

**New information:**

A new species *Chinattushuanggangshan* K. K. Liu, **sp. nov.** is reported from Jiangxi Administration Bureau in Wuyi Mountain National Park, Jiangxi Province. Detailed morphological descriptions, photographs and the distribution map of this species are provided.

## Introduction

The genus *Chinattus* Logunov, 1999 (Araneae, Salticidae, Hasariini), established by Logunov (1999) with *Heliophanusundulatus* Song & Chai, 1992 as the type species, currently comprises 20 recognised species (World Spider Catalog 2025). This genus exhibits a broad, yet disjunct distribution, predominantly spanning the south-eastern Palaearctic and adjacent Oriental Regions, including East Asia, the Himalayas and parts of Southeast Asia (Logunov 2021). Notably, *C.parvulus* (Banks, 1895) represents the sole Nearctic species (Edwards 2003), occurring in south-eastern Canada and the eastern United States, while the remaining taxa are confined to the Ancient Mediterranean and Palaearchearctic subregions, with a distinct Himalayan-Chinese distributional core (Logunov 2021, World Spider Catalog 2025). Despite moderate species diversity, taxonomic knowledge of *Chinattus* remains fragmentary: approximately 68% of its species are documented solely from type localities or original descriptions and no comprehensive revision exists to clarify interspecific boundaries or phylogenetic relationships (Logunov 2021). Recent discoveries from South and Southeast Asia (e.g. Nepal, India, Vietnam) highlight underestimated diversity, particularly in montane ecosystems, suggesting potential cryptic speciation and biogeographic complexity (Jastrzębski and Patoleta 2014, Basumatary et al. 2020, Logunov 2021).

Jiangxi Province, characterised by its classic hilly terrain, harbours exceptionally high biodiversity, reflecting the ecological complexity of such landscapes (Ji and Peng 2014). Accumulating data over recent years further corroborates the remarkable species richness and diversity endemic to these undulating regions (Liu et al. 2020, Liu et al. 2021, Liu et al. 2022a, Liu et al. 2022b, Liu et al. 2022c, Wang et al. 2024). This persistent pattern underscores the critical need for systematic investigation to unravel the ecological drivers, taxonomic composition and conservation priorities within these biodiverse habitats. The unique topographic heterogeneity of Jiangxi’s hills likely fosters niche diversification (Yao et al. 2021, Liu et al. 2022c) and microhabitat specialisation, making it a compelling focal area for biogeographic and taxonomic research.

While examining jumping spider specimens collected from the Wuyi Mountain National Park, Jiangxi Province, China, we identified a previously undescribed species. The aims of the present paper are to: (1) provide a comprehensive morphological description of this new taxon and (2) report novel distributional records for the genus within montane ecosystems, thereby expanding its known biogeographic range.

## Materials and methods

Specimens were examined using a Jiangnan SZ6100 stereomicroscope with a KUY NICE CCD camera. Male and female copulatory organs were dissected and examined in 80−85% ethanol. The endogynes were cleaned with pancreatin. All specimens were photographed with an Olympus CX43 compound microscope with a KUY NICE CCD camera.

All measurements were made using a stereomicroscope (AxioVision SE64 Rel. 4.8.3) and are given in millimetres. Leg measurements are given as total length (femur, patella, tibia, metatarsus, tarsus). The abbreviations used in the text are as follows: ALE − anterior lateral eye, AME − anterior median eye, BP – basal plate, CD − copulatory duct, CO − copulatory opening, d – dorsal, EB – embolic base, Em – embolus, ET – embolic tip, FD − fertilisation duct, GA − glandular appendage, p – prolateral, PL – posterior lobe, PLE − posterior lateral eye, PME − posterior median eye, r – retrolateral, RTA − retrolateral tibial apophysis, SD – sperm duct, Spe – spermatheca, v – ventral.

## Taxon treatments

### Chinattus huanggangshan

K. K. Liu sp. nov.

AB889D4E-6A01-580C-8EB0-5FC38F7B3681

4E178D01-6684-44A8-BFAA-EC0A1D1BCDFC

#### Materials

- **Type status:** Holotype. **Occurrence:** catalogNumber: Sal-417; recordedBy: Liu Ke-ke; individualCount: 1; sex: male; lifeStage: adult; occurrenceID: C001EB4D-3E81-568A-9DAF-37264D705A36; **Taxon:** scientificName: *Chinattushuanggangshan* **sp. nov.**; **Location:** country: China; stateProvince: Jiangxi; locality: Shangrao City, Yanshan County, Wuyi Mountain National Park, Tongmuguan Station; verbatimElevation: 1153m; verbatimCoordinates: 27°49'04.71"N, 117°42'53.27"E; georeferenceProtocol: GPS; **Event:** samplingProtocol: beating; eventDate: 7/9/2023
- **Type status:** Paratype. **Occurrence:** catalogNumber: Sal-417; recordedBy: Liu Ke-ke; individualCount: 1; sex: female; lifeStage: adult; occurrenceID: 6A1C0CF3-F96C-5AA5-97FE-4E164B987E0B; **Taxon:** scientificName: *Chinattushuanggangshan* **sp. nov.**; **Location:** country: China; stateProvince: Jiangxi; locality: Shangrao City, Yanshan County, Wuyi Mountain National Park, Tongmuguan Station; verbatimElevation: 1153m; verbatimCoordinates: 27°49'04.71"N, 117°42'53.27"E; georeferenceProtocol: GPS; **Event:** samplingProtocol: beating; eventDate: 7/9/2023

#### Description

**Figure 1.**
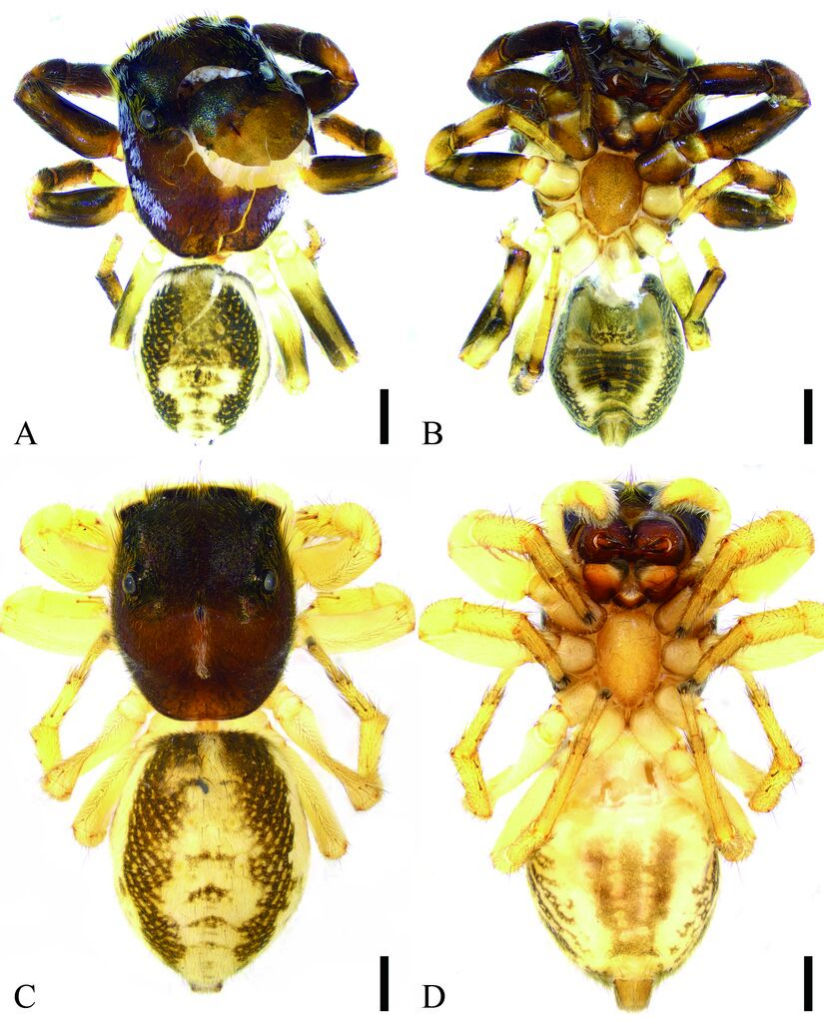
*Chinattushuanggangshan* sp. nov., habitus of male holotype and female paratype. **A** Male habitus, dorsal view; **B** Same, ventral view; **C** Female habitus, dorsal view; **D** Same, ventral view. Scale bars: 0.5 mm.

Male (holotype, Sal-417). Habitus (Fig. 1A, B). Total length 3.82. Carapace 2.09 long. Eye sizes and interdistances: AME 0.42, ALE 0.26, PME 0.09, PLE 0.20, AME−AME 0.18, AME−ALE 0.08, PME−PME 1.23, PME−PLE 0.16, AME−PME 0.52, AME−PLE 0.76, ALE−ALE 0.94, PLE−PLE 1.09, ALE−PLE 0.57. MOA length 0.84, anterior width 0.91, posterior width 1.33. Chelicerae (Fig. 1B) with two promarginal teeth and one retromarginal teeth. Sternum (Fig. 1B) shield-shaped, longer than wide, anterior margin nearly straight. Leg measurements: I 3.60 (1.10, 0.74, 0.79, 0.57, 0.40); II 2.83 (0.94, 0.57, 0.54, 0.49, 0.29); III 3.60 (1.25, 0.58, 0.66, 0.76, 0.35); IV 3.52 (1.08, 0.54, 0.71, 0.82, 0.37). Leg spination: I ti pv1-0-2, rv1-0-2; met pv1-0-1, rv1-0-1; II ti pv1-1-1, rv1-1-1; met pv1-0-1, rv1-0-1; III ti pr1-1-0, rt1-1-0, pv1-0-1, rv1-0-1; met pr1-0-1, rt1-0-1, pv1-0-1, rv1-0-1; IV ti pr1-0-0, rt1-0-0, pv1-0-0, rv1-0-0; met pr0-1-1, rt0-1-1, pv1-0-1, rv1-0-1. Pedicel 0.14. Abdomen 1.63 long, 1.24 wide.

Colouration (Fig. 1A, B). Carapace reddish-brown to dark brown dorsally, densely covered with white fine setae throughout; median pars cephalica bearing scale-like setae with greenish iridescence; eye bases encircled by black pigment; posterior median eyes medially positioned; fovea distinct, linear in form. Chelicerae reddish-brown. Endites yellowish-brown, length subequal to width, inner margins fringed with dense fine setae. Labium V-shaped. Sternum yellowish-brown, scutiform, anterior margin straight, densely speckled with yellow spots. Legs yellowish-brown to reddish-brown; leg I robust, dark brown. Abdomen dorsally displaying two pairs of distinct yellow muscle marks, lateral areas densely spotted with yellow markings, posterior portion with several white striations; venter showing corrugated striations and yellow linear markings laterally.

**Figure 2.**
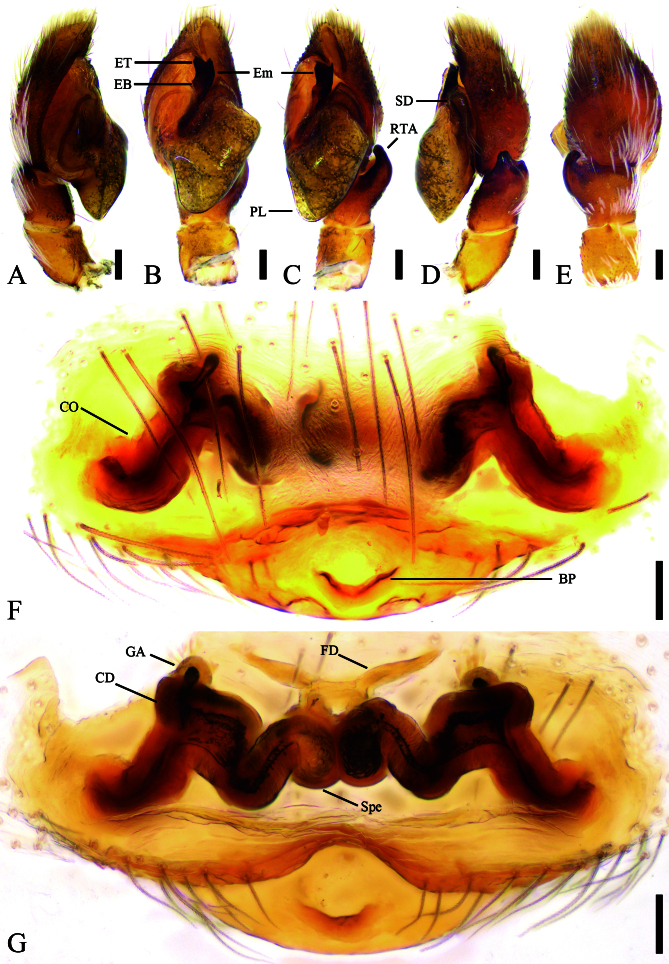
*Chinattushuanggangshan* sp. nov., male palp of holotype and female epigyne of paratype. **A** Palp, prolateral view; **B** Same, ventral view; **C** Same, ventral view, slightly retrolateral; **D** Same, retrolateral view; **E** Same, dorsal view; **F** Epigyne, ventral view; **G** Same, dorsal view. Abbreviations: BP – basal plate, CD – copulatory duct, CO − copulatory opening, EB – embolic base, Em – embolus, ET – embolic tip, FD − fertilisation duct; GA − glandular appendage, PL – posterior lobe, RTA – retrolateral tibial apophysis, SD – sperm duct, Spe – spermatheca. Scale bars: 0.1 mm (A–G).

Palp (Fig. 2A–E). Retrolateral tibial apophysis (RTA) thick and long, about 1/2 of tibia length, with a slightly curved tip in ventral view, stout in retrolateral view, hook-like in dorsal view. Cymbium about 1.5 times as long as wide. Bulb large and slightly swollen, nearly as long as cymbium, with broad posterior lobe (PL) extending postero-prolaterally. Sperm duct (SD) thick with right-angled bend proximal to the embolus base. Embolus (Em) strongly sclerotised, broad, with a broad tip and short retrolateral spine-like apophysis. Embolic base (EB) thick, originating at the medial part and terminating at the 2 o'clock position of the tegulum. Embolic tip (ET) bifurcated, Y-shaped.

**Female** (paratype, Sal-417). *Habitus* as in Fig. 1C, D. Total length 4.85. Carapace 2.26 long, 1.76 wide. Eye sizes and interdistances: AME 0.31, ALE 0.21, PME 0.08, PLE 0.13, AME−AME 0.16, AME−ALE 0.12, PME−PME 1.07, PME−PLE 0.21, AME−PME 0.46, AME−PLE 0.72, ALE−ALE 0.94, PLE−PLE 1.02, ALE−PLE 0.54. MOA: 0.71 long; 0.78 anterior width, 1.17 posterior width. Chelicerae (Fig. 1D) with two promarginal teeth and one retromarginal teeth. Sternum (Fig. 1D) oval, longer than wide, anterior margin arcuate. Leg measurements: I 2.80 (0.86, 0.57, 0.57, 0.47, 0.33); II 2.48 (0.80, 0.52, 0.48, 0.41, 0.27); III 2.74 (0.90, 0.47, 0.52, 0.61, 0.24); IV 3.19 (1.01, 0.54, 0.62, 0.72, 0.30). Leg spination: I ti pv1-1-1, rv1-1-1; met pr1-0-1, rt1-0-1, pv1-0-1, rv1-0-1; II ti pv1-1-1, rv1-1-1; met pr1-0-1, rt1-0-1; III ti pr0-1-1, rt0-1-1, pv1-0-0; met pr1-0-1, rt1-0-1; IV ti pv0-1-1; met pr1-0-1, rt1-0-1, pv0-0-1, rv0-0-1. Pedicel 0.06. Abdomen 2.5 long, 1.91 wide.

Colouration (Fig. 1C, D). Abdomen white centrally, other characteristics similar to male.

Epigyne (Fig. 2F, G). Epigynal plate ovoid. Basal plate (BP) with a median mastoid-like depression. Copulatory openings (CO) located laterally on epigynal plate. Copulatory ducts (CD) transversely extending, M-shaped, bearing the papillary glandular appendage (GA) dorsally. Spermathecae (Spe) pear-shaped, inner margin closely touching. Fertilisation ducts (FD) nearly twice as long as spermathecae, directed anterolaterally.

#### Diagnosis

The male is similar to that of *Chinattuswengnanensis* Cao & Li, 2016 (see Cao et al. (2016): 62, figs. 13A–D, 14C and E) in the strongly sclerotised and broad embolus with the bifurcated embolic tip, but can be distinguished from it by the diamond-shaped bulbus in ventral view (vs. triangular) (Fig. 2A–E), the tubular embolus with the Y-shaped apex (vs. beak-shaped) and the retrolateral tibial apophysis with robust base and blunt apex, gradually narrowing while curving inwards towards the cymbium (vs. digitiform, short and sharply pointed). The female resembles *C.tibialis* (Żabka, 1985) (see Peng (2020): 69, figs. 30e–f) in the oval epigynal plate with the basal apophysis, but differs by the M-shaped transverse copulatory ducts (vs. simply transversely extending) (Fig. 2F, G), the basal plate with an arcuate depression (vs. absent) and the short papillary glandular appendages (vs. elongated and bar-shaped).

#### Etymology

The specific name refers to the type locality that is the mountain peak (Huanggangshan) of the Wuyi Mountains; noun in apposition.

#### Distribution

**Figure 3.**
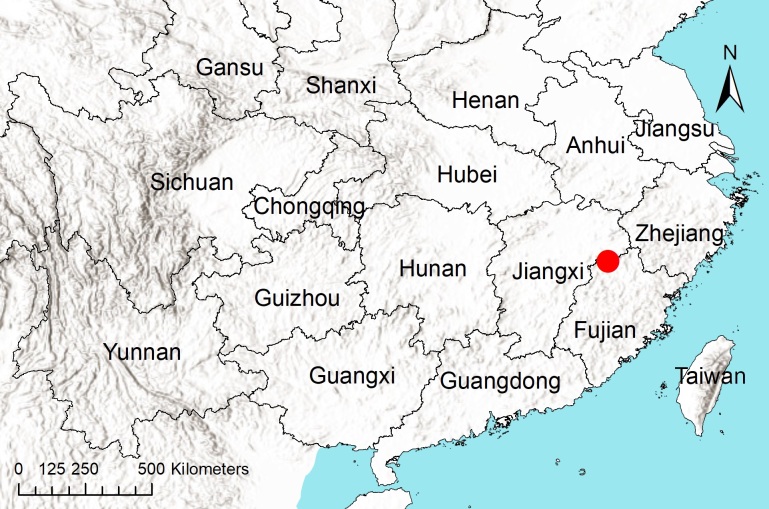
The location of the Jiangxi Region in Wuyi Mountain National Park, China indicated by a large red dot.

Known only from the type locality in Jiangxi Province, China (Fig. 3).

#### Ecology

It was collected in broad-leaved forests.

## Discussion

The discovery of *Chinattushuanggangshan* sp. nov. in Wuyi Mountain National Park, Jiangxi Province, holds significant implications for biodiversity research and conservation. First, this finding fills a critical geographical gap in the known distribution of the genus *Chinattus*, which had not been previously recorded in Jiangxi despite its presence in neighbouring provinces, such as Hunan and Fujian (Peng 2020). The species’ occurrence in this region underscores the ecological uniqueness of Wuyi Mountain, a biodiversity hotspot renowned for its rich endemic flora and fauna. Second, the discovery emphasises the role of protected areas like Wuyi Mountain National Park in conserving understudied arthropod groups. Similar to findings in beetle diversity of Nanling Priority Area (Ge et al. 2024, Yang 2024), this reinforces the necessity of systematic biodiversity surveys to uncover hidden species and refine conservation priorities. Finally, this discovery not only enriches China’s salticid species, but also highlights the urgency of preserving montane ecosystems amidst increasing anthropogenic pressures.

## Supplementary Material

XML Treatment for Chinattus huanggangshan
